# Metformin Impairs Mitochondrial Function in Skeletal Muscle of Both Lean and Diabetic Rats in a Dose-Dependent Manner

**DOI:** 10.1371/journal.pone.0100525

**Published:** 2014-06-20

**Authors:** Bart Wessels, Jolita Ciapaite, Nicole M. A. van den Broek, Klaas Nicolay, Jeanine J. Prompers

**Affiliations:** 1 Biomedical NMR, Department of Biomedical Engineering, Eindhoven University of Technology, Eindhoven, The Netherlands; 2 Department of Pediatrics, Centre for Liver, Digestive and Metabolic Diseases, University Medical Center Groningen, University of Groningen, Groningen, The Netherlands; Pennington Biomed Research Center, United States of America

## Abstract

Metformin is a widely prescribed drug for the treatment of type 2 diabetes. Previous studies have demonstrated *in vitro* that metformin specifically inhibits Complex I of the mitochondrial respiratory chain. This seems contraindicative since muscle mitochondrial dysfunction has been linked to the pathogenesis of type 2 diabetes. However, its significance for *in vivo* skeletal muscle mitochondrial function has yet to be elucidated. The aim of this study was to assess the effects of metformin on *in vivo* and *ex vivo* skeletal muscle mitochondrial function in a rat model of diabetes. Healthy *(fa/+)* and diabetic *(fa/fa*) Zucker diabetic fatty rats were treated by oral gavage with metformin dissolved in water (30, 100 or 300 mg/kg bodyweight/day) or water as a control for 2 weeks. After 2 weeks of treatment, muscle oxidative capacity was assessed *in vivo* using ^31^P magnetic resonance spectroscopy and *ex vivo* by measuring oxygen consumption in isolated mitochondria using high-resolution respirometry. Two weeks of treatment with metformin impaired *in vivo* muscle oxidative capacity in a dose-dependent manner, both in healthy and diabetic rats. Whereas a dosage of 30 mg/kg/day had no significant effect, *in vivo* oxidative capacity was 21% and 48% lower after metformin treatment at 100 and 300 mg/kg/day, respectively, independent of genotype. High-resolution respirometry measurements demonstrated a similar dose-dependent effect of metformin on *ex vivo* mitochondrial function. In conclusion, metformin compromises *in vivo* and *ex vivo* muscle oxidative capacity in Zucker diabetic fatty rats in a dose-dependent manner.

## Introduction

Metformin is the most commonly prescribed drug to treat type 2 diabetes and it has been in clinical use for decades. Metformin is a biguanide that lowers blood glucose levels primarily by improving insulin sensitivity in the liver, where it effectively inhibits gluconeogenesis [1], whereas it does not have marked hypoglycemic effects [2]. Moreover, metformin enhances insulin sensitivity in skeletal muscle, thereby stimulating peripheral glucose utilization [3].

A number of *in vitro* studies reported that metformin inhibits Complex I of the mitochondrial respiratory chain [3]–[9], thus limiting the respiratory capacity of the cell and possibly restricting ATP synthesis. The mechanism through which metformin acts on Complex I, however, is still not known. Some studies suggest that metformin binds directly to the mitochondrial membrane phospholipids, thereby altering physicochemical membrane properties [3], [10]. Others contradict this direct mechanism and postulate that an intact cell is required for metformin's inhibitory action on Complex I, involving an indirect pathway via the cell membrane [6], [8].

Treatment of patients with type 2 diabetes with a Complex I inhibitor seems contraindicative, since muscle mitochondrial dysfunction has been linked to the pathogenesis of this disease [11], [12]. Moreover, considering that regular exercise is recommended in most guidelines for the treatment of type 2 diabetes [13], [14], it seems even more unfavorable to treat diabetes patients with a Complex I inhibitor, as it would attenuate their exercise capacity and consequently their ability to increase insulin sensitivity via exercise training. The latter has indeed been demonstrated by Sharoff *et al.* [15], who observed that the therapeutic effects of exercise training were absent in patients who were treated with metformin in conjunction with exercise therapy.

Although the specific inhibitory action of metformin on Complex I has been shown using *in vitro* measurements, its significance for *in vivo* skeletal muscle mitochondrial function has yet to be elucidated. The aim of this study was to determine the effect of metformin on *in vivo* skeletal muscle oxidative capacity in a rat model of diabetes using phosphorous (^31^P) magnetic resonance spectroscopy (MRS). Lean, healthy and obese, diabetic Zucker diabetic fatty (ZDF) rats were dosed orally for 2 weeks with metformin dissolved in water (30, 100 or 300 mg/kg body weight/day) or water as a control. A dosage of 30 mg/kg/day is typically prescribed for diabetes patients, while, because of the lower bioavailability of metformin in rats compared with humans, ∼100–300 mg/kg/day metformin is needed to attain similar effects on glucose homeostasis in rats [3], [16]–[21]. ^31^P MRS measurements were complemented by *ex vivo* high resolution respirometry (HRR) measurements in isolated mitochondria to interpret the effects of metformin on *in vivo* oxidative capacity. We demonstrated that 2 weeks of treatment with metformin compromised *in vivo* and *ex vivo* muscle oxidative capacity in ZDF rats in a dose-dependent manner.

## Research Design and Methods

### Ethics statement

All experimental procedures were reviewed and approved by the Animal Experimental Committee of Maastricht University (permit number: 2011-047). Surgery, MRS experiments and termination were performed under isoflurane (IsoFlo) anesthesia with additional pain relief using buprenorphine (Temgesic), and all efforts were made to minimize suffering.

### Animals

Lean, non-diabetic *fa/+* and obese, diabetic, *fa/fa* adult male ZDF rats (12 weeks of age) were purchased from Charles River Laboratories (Sulzfield, Germany). The animals were housed pairwise, in a controlled environment (20°C and 50% relative humidity on a 12-h light-dark cycle) and given *ad libitum* access to water and specific standardized chow for ZDF rats (Purina Formula 5008, Bioservices, the Netherlands). For 15 days, animals were dosed with metformin (0, 30, 100 or 300 mg/kg body weight/day, n = 6 per group) in 1 ml of water directly into the stomach by oral gavage. Dosing was performed once daily between 4 and 6 pm. At day 15, *in vivo* MRS experiments were performed on the animals between 8 am and 4 pm, i.e. 14–24 hours after the dosage of metformin on the previous day. Following the MRS measurements, animals were administered with the last dose of metformin (between 4 and 6 pm). The following day between 8 and 10 am, i.e. 14–18 hours after the last dosage of metformin, animals were sacrificed under anesthesia by incision of the *vena cava*. The terminal half-life of metformin after oral administration in rats has been determined to be ∼3, 6 and 7 hours at doses of 50, 100 and 200 mg/kg, respectively [22], which implies that all *in vivo* and *ex vivo* experiments were performed under conditions in which plasma levels of metformin were less than 5% of the maximum plasma concentrations. One *tibialis anterior* (TA) muscle was used for isolation of mitochondria. The other TA was frozen in liquid nitrogen and stored at −80°C.

### Plasma parameters

After 2 weeks of treatment, a blood sample was taken between 12 and 2 pm (i.e. at least 18 hours after the previous dosage of metformin), after a 4-hour fast, for determination of post-therapy plasma glucose and insulin concentrations. Plasma glucose concentrations were determined using an automatic glucometer (Freestyle, Abbott, IL, USA). Plasma insulin concentrations were determined with an ultrasensitive rat insulin ELISA kit (Mercodia, Uppsala, Sweden).

### MRS measurements

^31^P MRS measurements were performed using a horizontal 6.3-T MR scanner (Bruker, Ettlingen, Germany) with an ellipsoid (10/18 mm) ^31^P surface coil. The animals were anaesthetized using isoflurane (2–3%) combined with medical air (0.6 L/min). ^31^P MRS was applied to assess *in vivo* oxidative capacity of the TA muscle, as described previously [23]. A fully relaxed spectrum (repetition time  =  20 s, 32 averages) was recorded first, followed by a time series of spectra (repetition time  =  5 s, 4 averages) obtained during a resting period of 3 min, 2 min of electrical stimulation and 15 min of recovery. Electrodes were implanted subcutaneously along the distal nerve trajectory of the *N. peroneus communis* to electrically stimulate the TA muscle. Pulses with a stimulation voltage of approximately 3 V were used to reach similar levels of phosphocreatine (PCr) depletion for the different animals.

### MRS data analysis

MR spectra were fitted in the time domain using a nonlinear least squares algorithm (advanced method for accurate, robust, and efficient spectral fitting; AMARES) in the jMRUI software package [24] as described previously [23]. In short, spectral analysis of the ^31^P MR spectra was done by fitting the PCr peak to Lorentzian and the inorganic phosphate (Pi) as well as the α-, β- and γ-ATP peaks to Gaussian line shapes. Intracellular pH was calculated from the chemical shift difference between the Pi and PCr resonances [25]. For the time series, the concentrations of PCr determined during recovery were fit to a mono-exponential function using Matlab (version 7.11.0, Mathworks, Natick, MA, USA) yielding a rate constant, *k*_PCr_, which is a measure of skeletal muscle mitochondrial oxidative capacity. For each rat, results from two time series with end-stimulation pH values higher than 6.9 were averaged [26].

### Determination of the relative mitochondrial DNA copy number

The relative mitochondrial-DNA copy number was measured as described previously [27]. Briefly, genomic DNA was isolated from a 25 mg transversal slice of mid-belly TA using GenElute Mammalian Genomic DNA Miniprep Kit (Sigma-Aldrich, Zwijndrecht, The Netherlands). Mitochondrial DNA (mtDNA) content relative to peroxisome proliferator-activated receptor-γ coactivator 1α (PGC-1α) gene was measured using real-time PCR as described in [28].

### High-resolution respirometry

Skeletal muscle mitochondria were isolated from whole TA muscle through a differential centrifugation procedure as described elsewhere [27]. Mitochondrial protein content was determined using a BCA protein assay kit (Pierce, Thermo Fisher Scientific Inc., Rockfort, IL, USA). *Ex vivo* mitochondrial function was evaluated by measuring oxygen consumption rates (O_2_ flux) at 37°C using a 2-channel high-resolution Oroboros oxygraph-2k (Oroboros, Innsbruck, Austria) as described previously [27]. O_2_ flux was fueled either with 5 mM pyruvate plus 5 mM malate (Complex I respiration) or 5 mM succinate plus 1 µM rotenone (Complex II respiration). Maximal rates of oxygen consumption coupled to ATP synthesis, i.e. the OXPHOS state (classical state 3), was determined after addition of an ADP-regenerating system consisting of excess hexokinase (4.8 U/ml), glucose (12.5 mM) and ATP (1 mM). The resting state respiration, which compensates for proton leak, i.e. the LEAK state (classical state 4), was assessed after addition of 1.25 µM carboxyatractyloside (CAT). Finally, the maximal capacity of the electron transfer system (ETS), i.e. the ETS state (classical state U), was determined by uncoupling the ETS from ATP synthesis with the addition of 1 µM carbonyl cyanide 3-chlorophenyl hydrazone (CCCP) [29]. The respiratory control ratio (RCR) was calculated as the ratio of OXPHOS to LEAK states.

### High-resolution respirometry after in vitro incubation with metformin

Isolated mitochondria from a cohort of water-treated lean and diabetic ZDF rats (n = 5 per genotype) were incubated in assay medium supplemented with metformin (1 mM) for 5 minutes in the presence of pyruvate and malate or succinate plus rotenone (at 37°C), after which mitochondrial respiratory capacity was assessed in the OXPHOS state. Results were expressed relative to the oxygen consumption rates measured without incubation with metformin.

For all HRR measurements, signals from the oxygen electrode were recorded at 0.5-s intervals and measurements were done in duplicate. Data acquisition and analysis was performed using Oxygraph-2k-Datlab 4.3.1.15 software (Oroboros, Innsbruck, Austria).

### Statistical analysis

Data are presented as means ± SD. Statistical significance of genotype and treatment effects were assessed by applying a two-way Analysis of Variance (ANOVA) in the IBM SPSS 20 statistical package (SPSS Inc., Chicago, IL, USA). In case of a significant effect of treatment, Bonferroni corrected post-hoc tests were carried out in order to identify differences between different treatment regimens. In case the interaction between genotype and treatment was significant or borderline significant (P<0.1), the differences were evaluated in more detail by separately analyzing the effects of genotype and treatment using Bonferroni-corrected two-sided unpaired t-tests. For determination of mitochondrial respiratory capacity changes after *in vitro* incubation of mitochondria with metformin, statistical analysis was done using a 2×2 mixed design ANOVA with one within-subjects factor (metformin incubation) and one between-subjects factor (genotype) in SPSS. The level of statistical significance was set at P<0.05.

## Results

### Animal characteristics

Animal characteristics after 2 weeks of treatment are summarized in Table 1. Body weight was significantly higher in diabetic animals compared with lean animals (P<0.01), except for the water-treated groups (for which body weight also did not differ before start of treatment). Fasting plasma glucose (P<0.001) and insulin (P<0.01) were significantly higher in diabetic animals compared with lean animals, independent of treatment regimen. Two weeks of treatment with 30, 100 or 300 mg/kg/day metformin had no effect on body weight, fasting plasma glucose, or fasting plasma insulin in lean or diabetic animals.

**Table 1:** Animal characteristics of lean and diabetic ZDF rats after 2 weeks of treatment with water or 30, 100 or 300 mg/kg body weight/day metformin (MET30, MET100 and MET300, respectively).

	Body weight (g)	Fasting glucose (mM)	Fasting insulin (pM)
Lean			
Water	366±11	4.0±0.6	297±99
MET30	360±18	4.9±1.2	205±63
MET100	337±31	4.1±0.3	213±60
MET300	341±23	4.4±1.1	184±42
Diabetic			
Water	380±17	14.2±1.2	240±50
MET30	406±11^##^	13.2±5.5	329±128
MET100	386±15^###^	14.7±1.7	402±166
MET300	409±27^###^	15.1±0.8	440±164

Data is represented as mean ± SD (n = 6 per group). Fasting plasma glucose (ANOVA: P<0.001) and insulin (ANOVA: P<0.01) were significantly higher in diabetic animals compared with lean animals, independent of treatment regimen. For body weight, the interaction between genotype and treatment was significant and a pairwise analysis of differences is provided by Bonferroni-corrected two-sided unpaired t-tests: ^##^ P<0.01, ^###^ P<0.001 when compared with lean animals of the same treatment regimen.

### *In vivo* muscle mitochondrial oxidative capacity

**Figure 1:**
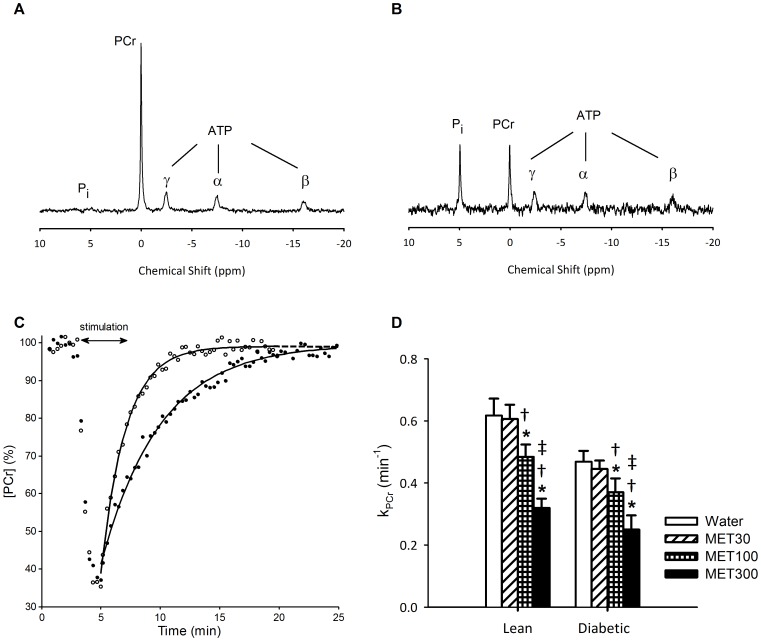
*In vivo* oxidative capacity of *tibialis anterior* (TA) muscle, assessed by ^31^P MRS. Representative examples of ^31^P MR spectra obtained during rest with 32 averages (A) and at the end of the electrical-stimulation protocol with 4 averages (B). (C) Representative examples of relative PCr concentrations during rest, muscle stimulation and recovery (time resolution  =  20 s) for a water-treated diabetic rat (open symbols) and a diabetic rat treated with metformin at 300 mg/kg body weight/day (filled symbols). PCr concentrations are expressed as a percentage of the resting PCr concentration. Mono-exponential functions (dark lines) were fit to the recovery data and the PCr recovery rate constants were 0.63 and 0.21 min^-1^ for the water-treated and metformin-treated animal, respectively. (D) Rate constants of PCr recovery, *k*_PCr_, after electrical stimulation in TA muscle of lean and diabetic rats treated with water or 30, 100 or 300 mg/kg body weight/day metformin (MET30, MET100 and MET300 respectively). Data is represented as mean ± SD (n = 6 per group). *k*_PCr_ was significantly lower in diabetic rats compared with lean rats, independent of treatment regimen (ANOVA: P<0.001). In addition, treatment had a significant effect on *k*_PCr_, independent of genotype, and a pairwise analysis of differences is provided by Bonferroni-corrected post-hoc tests: ^*^ P<0.001 when compared with water-treated animals, ^†^ P<0.001 when compared with MET30-treated animals, ^‡^ P<0.001 when compared with MET100-treated animals.

^31^P MRS was applied to assess the effect of metformin treatment on *in vivo* mitochondrial oxidative capacity. Representative examples of ^31^P MR spectra obtained from TA muscle at rest and after 2 minutes of electrical stimulation are shown in Figure 1A and 1B, respectively. PCr and Pi concentrations and intracellular pH measured in TA muscle at rest and after muscle stimulation are listed in Table 2. End-stimulation pH was significantly higher in diabetic animals compared with lean animals (P<0.01), independent of treatment regimen. However, the end-stimulation pH was higher than 7.0 for all animals and therefore did not influence PCr recovery kinetics. A mono-exponential function was fitted through the PCr concentrations obtained during the recovery phase (Figure 1C), yielding the PCr recovery rate constant, *k*_PCr_, which is representative for muscle oxidative capacity *in vivo*. *k*_PCr_ was 25% lower in diabetic rats compared with lean rats, independent of treatment regimen (P<0.001) (Figure 1D). Two weeks of treatment with metformin had a significant effect on in *in vivo* muscle oxidative capacity, independent of genotype (P<0.001). Post-hoc testing revealed that treatment of lean and diabetic rats with 30 mg/kg/day metformin did not affect *in vivo* muscle oxidative capacity when compared with water-treated controls. However, in rats treated with metformin at a dosage of 100 and 300 mg/kg/day, *in vivo* muscle oxidative capacity was 21% (P<0.001) and 47% (P<0.001) lower, respectively, when compared with water-treated animals.

**Table 2:** Metabolite concentrations and pH in TA muscle measured by ^31^P MRS of lean and diabetic ZDF rats after 2 weeks of treatment with water or 30, 100 or 300 mg/kg body weight/day metformin (MET30, MET100 and MET300, respectively).

	REST			END-STIMULATION			
	pH (−)	[PCr] (mM)	[Pi] (mM)	pH (−)	[PCr] (mM)	[Pi] (mM)	ΔPCr (%)
Lean							
Water	7.17±0.02	34.2±1.5	2.4±0.2	7.05±0.02	13.7±1.2	21.8±2.4	62.3±3.5
MET30	7.18±0.01	35.4±2.2	2.4±0.3	7.07±0.01	13.5±1.7	23.3±3.8	64.3±3.3
MET100	7.17±0.01	35.4±1.2	2.5±0.1	7.06±0.04	14.3±2.1	23.4±1.7	62.0±4.7
MET300	7.17±0.02	35.1±1.7	2.3±0.5	7.01±0.07	12.9±1.7	21.5±2.2	63.2±4.7
Diabetic							
Water	7.16±0.01	34.9±2.1	2.9±0.4	7.07±0.05	13.7±1.2	25.1±2.2^#^	63.7±3.1
MET30	7.16±0.03	36.0±2.7	2.5±0.4	7.13±0.03	16.1±1.2^##,*^	21.3±0.7	56.3±3.4^##^
MET100	7.17±0.02	35.3±2.0	2.8±0.6	7.09±0.02	13.4±1.6^†^	27.5±3.6^#,††^	65.6±3.8
MET300	7.15±0.01	33.8±1.8	2.5±0.6	7.08±0.08	14.3±1.6	25.5±2.6^#^	62.2±4.6

Data is represented as mean ± SD (n = 6 per group). At rest, pH was significantly lower and [Pi] was significantly higher in diabetic animals compared with lean animals, independent of treatment regimen (ANOVA: P<0.05). At the end of stimulation, pH was significantly higher in diabetic animals compared with lean animals, independent of treatment regimen (ANOVA: P<0.01). For end-stimulation [PCr], [Pi] and ΔPCr, the interaction between genotype and treatment was significant and a pairwise analysis of differences is provided by Bonferroni-corrected two-sided unpaired t-tests: * P<0.05 when compared with water-treated animals of the same genotype, ^#^ P<0.05, ^##^ P<0.01 when compared with lean animals of the same treatment regimen, ^†^ P<0.05, ^††^ P<0.01 when compared with MET30-treated animals of the same genotype.

### Mitochondrial content

**Figure 2:**
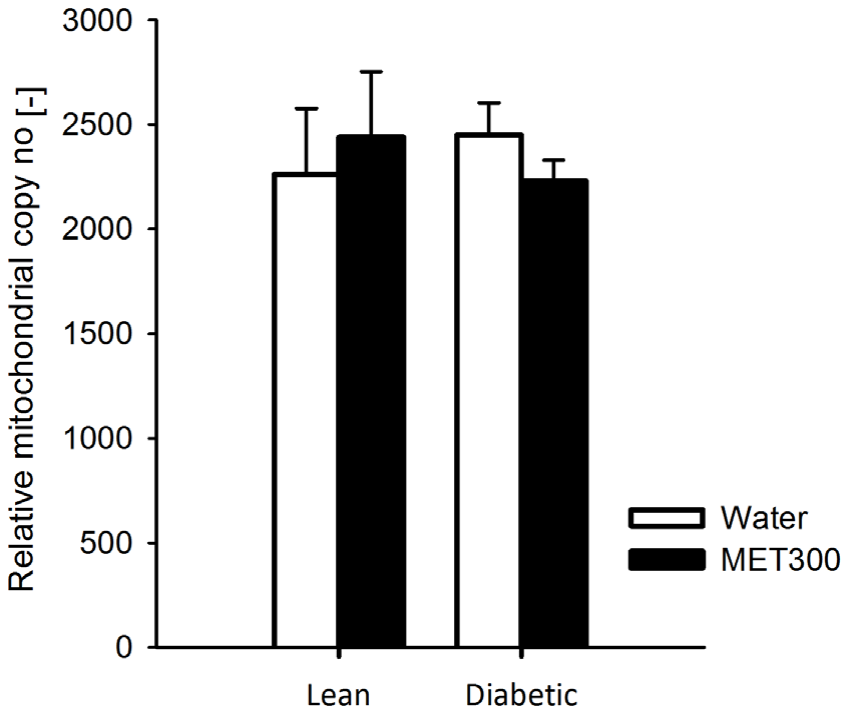
Relative mitochondrial-DNA copy number of lean and diabetic rats after 2 weeks of treatment with either water or metformin (300 mg/kg bodyweight/day). Data is represented as mean ± SD (n = 6 per group).

Skeletal muscle oxidative capacity is determined by intrinsic mitochondrial properties, as well as the number of mitochondria in the tissue. Relative mtDNA copy number, which was used as an estimate of mitochondrial content, did not differ between lean and diabetic rats (Figure 2). Moreover, metformin treatment (300 mg/kg/day) did not affect relative mtDNA copy number.

### *Ex vivo* mitochondrial function

In order to evaluate *ex vivo* intrinsic mitochondrial function after 2 weeks of oral treatment with metformin, HRR was used to measure O_2_ flux in mitochondria isolated from TA muscle, using both Complex I- and Complex II-dependent substrates.

#### Complex I

**Figure 3:**
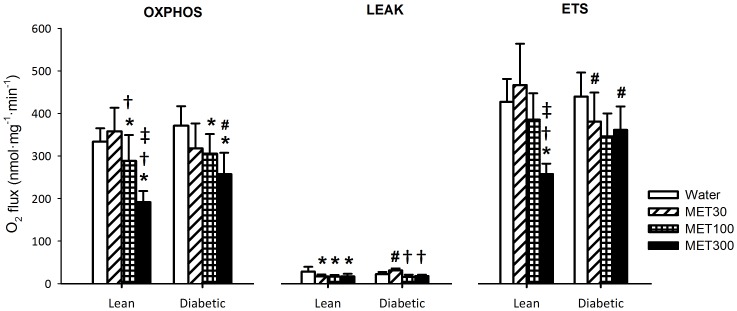
O_2_ consumption rates determined in mitochondria isolated from TA muscle of lean and diabetic rats treated with water or 30, 100 or 300 mg/kg body weight/day metformin (MET30, MET100 and MET300, respectively) for 2 weeks, fueled by pyruvate plus malate (Complex I-dependent substrate). Respiratory capacity was determined in the OXPHOS state, when mitochondrial respiration is coupled to ATP synthesis; the LEAK-state, when the system is limited by ADP; and the ETS state, after uncoupling of the ETS from ATP synthesis. Data is represented as mean ± SD (n = 6 per group). For the OXPHOS state, the interaction between genotype and treatment was borderline significant and for the LEAK and ETS state, the interaction between genotype and treatment was significant. A pairwise analysis of differences is provided by Bonferroni-corrected two-sided unpaired t-tests: ^*^ P<0.05 when compared with water-treated animals of the same genotype, ^†^ P<0.05 when compared with MET30-treated animals of the same genotype, ^‡^ P<0.05 when compared with MET100-treated animals of the same genotype, ^#^ P<0.05 when compared with lean animals of the same treatment regimen.

Complex I-dependent respiratory capacity (driven by pyruvate plus malate) in the OXPHOS state was not different between lean and diabetic animals, except at the highest metformin dosage (300 mg/kg/day), for which OXPHOS respiratory capacity was lower in lean rats compared with diabetic rats (P<0.05) (Figure 3). Whereas 2 weeks of treatment with metformin at 30 mg/kg/day did not affect Complex I-dependent respiratory capacity in the OXPHOS state, treatment at a dosage of 100 and 300 mg/kg/day lowered OXPHOS respiratory capacity compared with water treatment in both lean and diabetic animals (P<0.05). In lean animals, Complex I-dependent OXPHOS respiratory capacity was further reduced after metformin treatment at 300 mg/kg/day compared with 100 mg/kg/day (P<0.01), but this dose-dependent effect was not significant in diabetic animals. In lean animals, Complex I-dependent respiration in the LEAK state was lower after metformin treatment when compared with water treatment, for all metformin dosages (P<0.05) (Figure 3). As a consequence of the concomitant changes in OXPHOS and LEAK states in response to metformin treatment, the RCR's, which give an indication of the coupling efficiency between substrate oxidation and ATP synthesis, were not affected in lean animals (Table 3). In diabetic rats, the LEAK state was lower in the MET100 and MET300 groups compared with the MET30 group only (P<0.001) and the RCR was higher in the MET100 compared with MET30 group (P<0.05). In addition, the RCR was higher in water-treated diabetic rats compared with water-treated lean rats (P<0.05). In lean animals, treatment with metformin at a dosage of 300 mg/kg/day lowered Complex I-dependent respiratory capacity in the ETS state when compared to all other treatment regimens (P<0.01) (Figure 3). In contrast, metformin treatment had no significant effect on Complex I-dependent ETS respiratory capacity in diabetic rats.

**Table 3:** Respiratory control ratios (RCR's) in mitochondria isolated from TA muscle of lean and diabetic rats treated with water or 30, 100 or 300 mg/kg body weight/day metformin (MET30, MET100 and MET300, respectively) for 2 weeks, fueled by pyruvate plus malate (Complex I-dependent substrate) and succinate plus rotenone (Complex II-dependent substrate).

	RCR Pyruvate (−)	RCRSuccinate (−)
Lean		
Water	11.0±5.1	3.9±0.4
MET30	15.7±6.7	4.3±1.3
MET100	18.0±3.5	4.6±0.8
MET300	11.4±4.2	4.5±0.7
Diabetic		
Water	17.6±4.7^#^	4.2±0.6
MET30	10.4±2.3	4.4±1.0
MET100	20.1±6.9^†^	4.8±0.6
MET300	15.5±1.4	4.4±0.7

Data is represented as mean ± SD (n = 6 per group). For the RCR with pyruvate, the interaction between genotype and treatment was significant and a pairwise analysis of differences is provided by Bonferroni-corrected two-sided unpaired t-tests: ^#^ P<0.05 when compared with lean animals of the same treatment regimen,^†^ P<0.05 when compared with MET30-treated animals of the same genotype.

#### Complex II

**Figure 4:**
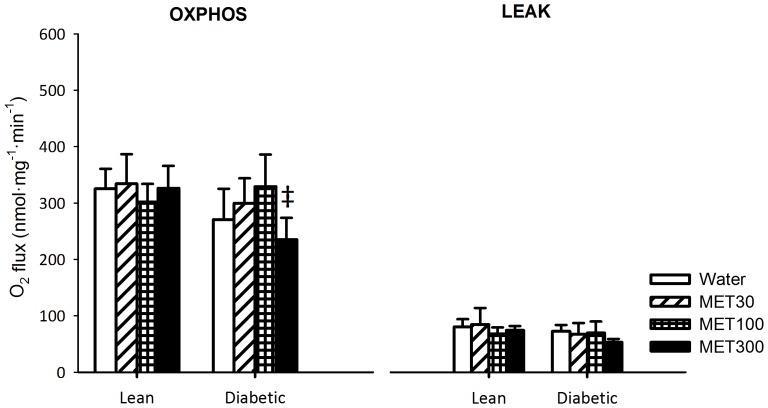
O_2_ consumption rates determined in mitochondria isolated from TA muscle of lean and diabetic rats treated with water or 30, 100 or 300 mg/kg body weight/day metformin (MET30, MET100 and MET300 respectively) for 2 weeks, fueled by succinate plus rotenone (Complex II-dependent substrate). Respiratory capacity was determined in the OXPHOS state, when mitochondrial respiration is coupled to ATP synthesis; and the LEAK-state, when the system is limited by ADP. Data is represented as mean ± SD (n = 6 per group). For the OXPHOS state, the interaction between genotype and treatment was significant and a pairwise analysis of differences is provided by Bonferroni-corrected two-sided unpaired t-tests: ^‡^ P<0.05 when compared with MET100-treated animals of the same genotype.

Complex II-dependent respiratory capacity (driven by succinate plus rotenone) in the OXPHOS state was not different between lean and diabetic animals (Figure 4). Moreover, treatment with metformin had no effect on Complex II-dependent OXPHOS respiratory capacity, except for diabetic rats treated with 300 mg/kg/day metformin, for which OXPHOS respiratory capacity was lower than for diabetic rats treated with 100 mg/kg/day metformin (P<0.01). Complex II-dependent respiration in the LEAK state (Figure 4) and RCR's (Table 3) were not different between groups.

### Mitochondrial function after in vitro incubation with metformin

**Figure 5:**
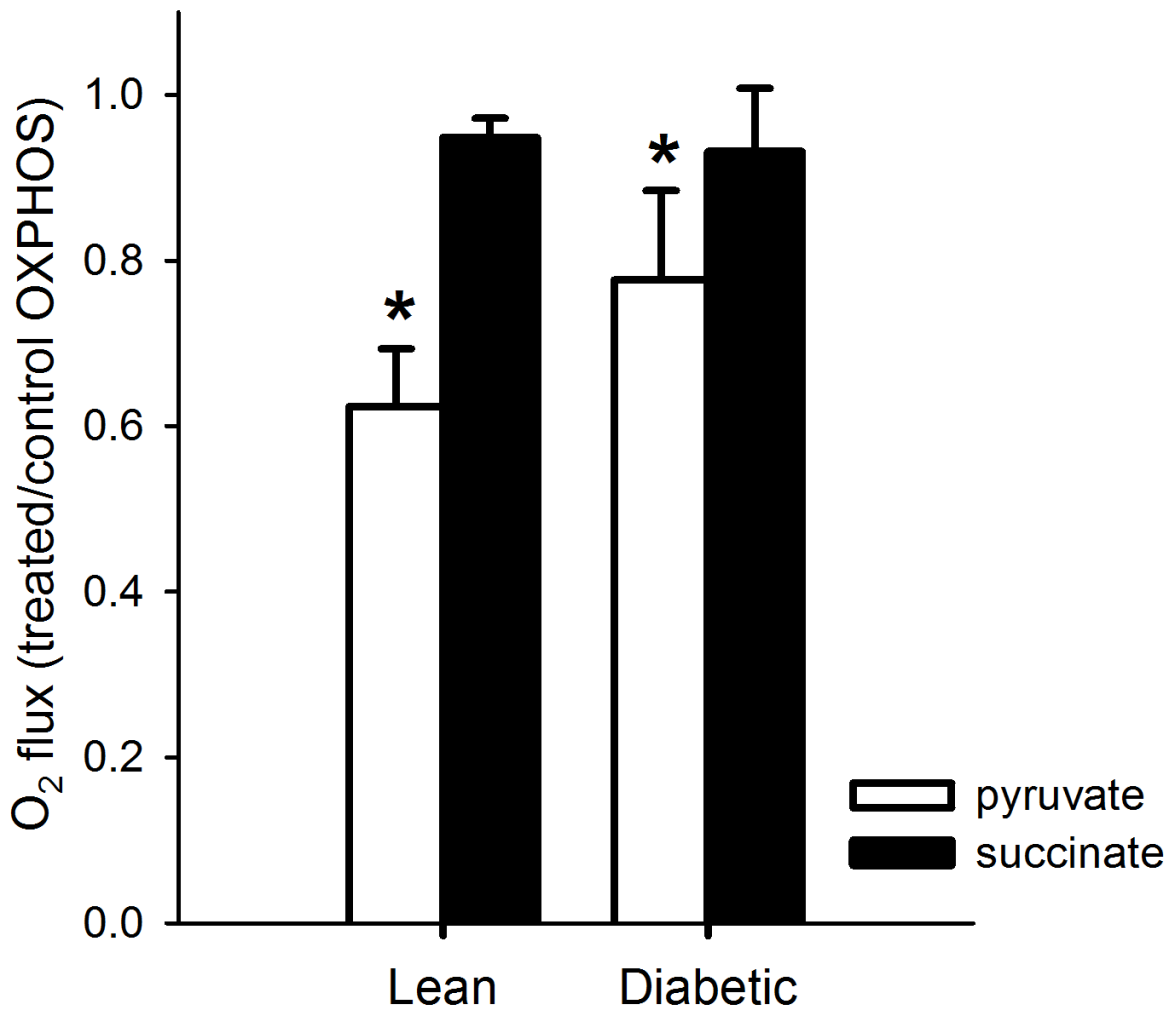
O_2_ flux measured in mitochondria isolated from TA muscle of lean and diabetic ZDF rats after 5 min of incubation with metformin (1 mM), normalized to O_2_ flux measured in isolated mitochondria without addition of metformin. Respiratory capacity was determined in the OXPHOS state, when mitochondrial respiration is coupled to ATP synthesis, fueled with either pyruvate plus malate (Complex I respiration) or succinate plus rotenone (Complex II respiration). Data is represented as mean ± SD (n = 6 per group). Incubation with metformin significantly lowered OXPHOS respiration fueled with pyruvate plus malate, independent of genotype (ANOVA: * P<0.001). Metformin did not affect Complex II respiration.

In order to assess whether metformin would affect mitochondrial respiratory capacity *in vitro*, mitochondria were isolated from TA muscle excised from lean and diabetic rats, and incubated with 1 mM metformin for 5 min. Complex I- and Complex II-dependent OXPHOS respiratory capacity were then determined and normalized to OXPHOS respiratory capacity measured in the isolated mitochondria without addition of metformin (Figure 5). Complex I-dependent respiratory capacity in the OXPHOS state decreased 28% after *in vitro* incubation with metformin, independent of genotype (P<0.001). In contrast, incubation of isolated mitochondria with metformin did not affect Complex II-dependent respiratory capacity.

## Discussion

A number of *in vitro* studies have shown that metformin inhibits Complex I of the mitochondrial respiratory chain [3]–[9]. However, the significance of this inhibition for *in vivo* skeletal muscle mitochondrial function has yet to be elucidated. The aim of this study was to clarify to which extent metformin affects *in vivo* and *ex vivo* skeletal muscle oxidative capacity. To this end we assessed the mitochondrial response to 2 weeks of treatment with metformin (0, 30, 100 or 300 mg/kg body weight/day) in a rat model of diabetes using ^31^P MRS and HRR, respectively. We showed that 2 weeks of treatment with metformin impairs *in vivo* muscle oxidative capacity in a dose-dependent manner, both in healthy and in diabetic rats. Whereas a dosage of 30 mg/kg/day had no significant effect, *in vivo* oxidative capacity was 21% and 48% lower after 2 weeks of metformin treatment at 100 and 300 mg/kg/day, respectively, independent of genotype. HRR measurements demonstrated a similar dose-dependent effect of metformin on *ex vivo* respiratory capacity with a Complex I-dependent substrate, whereas Complex II-dependent respiratory capacity was largely unaffected.

In contrast to the current belief that metformin has only a mild effect on mitochondrial function [30], we observed that metformin may severely impair skeletal muscle oxidative capacity *in vivo*, depending on the dosage. Two weeks of metformin treatment at 300 mg/kg/day led to a 2-fold reduction in the rate of PCr recovery after muscle stimulation, both in lean and diabetic rats, which is comparable to the 40% lower PCr recovery rate found in sedentary individuals as compared with endurance athletes, who run a minimum of 30 miles per week [31]. At 100 mg/kg/day, metformin had a more moderate effect on *in vivo* muscle oxidative capacity, while at 30 mg/kg/day no significant effect on PCr recovery was observed. Patients with type 2 diabetes typically receive an oral dose of metformin of approximately 30 mg/kg/day. It should be noted though that the bioavailability of metformin in the systemic circulation after oral treatment is lower in rats (F = 30%, [22]) compared with patients (F = 56%, [32]). Therefore treatment with ∼100–300 mg/kg/day metformin in rats is considered to be more clinically relevant, also because therapeutic effects of metformin treatment in rats at that dosage are similar to the effects in patients treated with 30 mg/kg/day metformin [3], [16]–[21]. It thus seems likely that patients with type 2 diabetes, possibly already featuring some level of mitochondrial impairment, will be affected in daily life functioning or when performing exercise as a consequence of treatment with metformin. Interestingly, Braun *et al.* observed a small but significant (2.7%) decrease in whole-body peak aerobic capacity (peak VO_2_) in healthy volunteers after 9–12 days of treatment with 2000 mg/day (which equals 30 mg/kg body weight/day) metformin [30]. Moreover, a study by Sharoff *et al*. demonstrated that exercise-induced improvement of whole-body insulin sensitivity is lost in insulin-resistant individuals taking metformin [15]. Their findings essentially imply that metformin treatment in these patients limits their ability to improve their peripheral insulin sensitivity through exercise.

*In vivo* skeletal muscle oxidative capacity is determined by intrinsic mitochondrial function as well as mitochondrial content. However, we did not find a difference in relative mtDNA copy number or PGC-1α protein expression (not shown) between rats treated with metformin (300 mg/kg/day) and water-treated controls, which implies that the observed reduction in *in vivo* skeletal muscle oxidative capacity after metformin treatment is not caused by a decrease in mitochondrial content. In fact, Suwa *et al*. [33] reported enhanced protein expression of PGC-1α and increased citrate synthase activity in Wistar rats after 2 weeks of treatment with metformin, suggesting a stimulation of mitochondrial biogenesis. However, the dosage regimen used in that study was twice as high as the highest dosage used in the present study, which might explain why we did not observe an effect on mitochondrial biogenesis. Our results are in agreement with other rodent studies showing that 2 or 4 weeks of metformin treatment at ∼300 mg/kg/day does not lead to increased activity of citrate synthase [21], [34].

In order to evaluate intrinsic mitochondrial function, we performed HRR measurements in isolated muscle mitochondria from rats treated with metformin using both Complex I- and Complex II-dependent substrates. Two weeks of treatment with metformin affected Complex I-dependent respiratory capacity in the OXPHOS state, similar to the dose-dependent effect observed for *in vivo* muscle oxidative capacity. At 300 mg/kg/day, Complex I-dependent respiratory capacity in the OXPHOS state was ∼40% lower than in water-treated controls, which is comparable to the 48% reduction in *in vivo* muscle oxidative capacity. For lean rats, the effect of metformin on Complex I-dependent respiratory capacity in the OXPHOS state was similar to that in the ETS state, indicating that the effect of metformin is confined to the respiratory chain. Moreover, metformin did not increase respiration in the LEAK state. Therefore, it seems that the effect of metformin on *in vivo* muscle oxidative capacity can be fully explained by its inhibition of Complex I-dependent respiration. The inhibitory action of metformin on Complex I-dependent respiration has been demonstrated before in *in vitro* studies, in which isolated mitochondria from rat liver [3]–[5] and skeletal muscle [4], as well as permeabilized cells [3], [6], [7] were incubated with metformin. Reports on Complex I activity in cultured cells further support an inhibitory effect of metformin on Complex I [4], [7]. In contrast, other *ex vivo* animal studies reported no effects on the respiratory capacity of permeabilized muscle fibers obtained from the oxidative part of the *gastrocnemius* of obese Zucker rats after 4 weeks of treatment with metformin (320 mg/kg/day) [34] and the predominantly glycolytic TA of wild type mice after 2 weeks of treatment with metformin (300 mg/kg/day) [21]. Likewise, it was shown that in permeabilized *vastus lateralis* muscle fibers of type 2 diabetes patients treated with metformin (2000±200 mg/day) Complex I-dependent respiratory capacity was not different compared with healthy control subjects, indicating that mitochondrial Complex I respiration is not inhibited by metformin [35]. Surprisingly, in L6 muscle cell cultures [36] and in skeletal muscle of kinase dead AMPK mice [21] metformin even increased mitochondrial energy formation. The discrepancies across the literature could be caused by differences in species, dosing regimens, muscle fiber types, and the methods used to determine the effect of metformin on the mitochondria. However, when comparing our results with the *ex vivo* animal studies of Kane et al. [34] and Kristensen et al. [21], in which metformin did not affect mitochondrial respiration in either oxidative or glycolytic muscle from rats or mice after 2–4 weeks of metformin treatment at ∼300 mg/kg/day, it seems that all except methodological differences can be excluded. In the current study mitochondria were isolated from a whole TA muscle to allow comparison with the *in vivo* data, while Kane et al. and Kristensen et al. used permeabilized muscle fibers. It has recently been reported that the respiratory response in permeabilized fibers can be different from that of isolated mitochondria [37].

In this study, Complex II-dependent OXPHOS respiratory capacity was largely unaffected by metformin treatment. This is in agreement with previous reports showing that metformin has no effect on Complex II-dependent respiratory capacity [4], [6]. Schäfer and Rieger postulated that metformin inhibits the activity of the oxidative phosphorylation enzymes by binding to the mitochondrial membrane phospholipids and modifying physicochemical membrane properties [10]. Following this reasoning, it is not surprising that the activity of Complex I, the largest and most complex enzyme among the enzymes involved in the oxidative phosphorylation pathway, is impaired the most by metformin. Our observation that Complex II-dependent OXPHOS capacity in mitochondria from diabetic rats treated with 300 mg/kg/day metformin was lower than for diabetic rats treated with 100 mg/kg/day metformin suggests that the activity of Complex II or of downstream electron transport chain complexes (i.e Complex III and/or IV) is impaired by a high dosage of metformin. This inhibitory effect could be partially caused by a progressively larger derangement of the inner mitochondrial membrane by a high concentration of metformin [10], impairing the activity of smaller ETC complexes. Possibly, mitochondria from skeletal muscle of diabetic animals are more sensitive to the toxic metformin effect due to other factors related to the diseased environment, since we do not observe inhibition of Complex II-dependent respiration in mitochondria from lean animals. However, our results on Complex I-dependent respiration do not support the notion that mitochondria from diabetic muscle are more sensitive to metformin-induced membrane derangements.

Although it is well established that metformin attenuates Complex I-dependent respiratory capacity, the mechanism through which metformin exerts its inhibitory action on Complex I is still subject of debate. A number of reports propose an indirect pathway, involving cell membrane events, via which metformin affects mitochondrial respiration [6], [8]. This is based on the observation that inhibition of Complex I is lost when metformin is added to mitochondria isolated from their cellular environment [6] or when metformin is micro-injected into the interior of an intact oocyte, suggesting membrane-mediated events are necessary for this effect to occur [6], [8]. Others, however, have shown that metformin does inhibit Complex I in mitochondria isolated from skeletal muscle and liver [3], [4], thus contradicting the suggestion that an intact cell is needed for metformin to exert its effect on mitochondrial function. Early work of Schäfer and Rieger [10] showed that biguanides have an affinity to directly bind to mitochondrial membrane phospholipids, causing the accumulation of positive charge at the membrane surface, thereby rendering the electrostatic surface potential more positive. This will alter the physicochemical properties of the mitochondrial membrane, which may underlie the inhibition of metformin of Complex I and which supports a direct pathway for metformin to affect mitochondria. In order to determine whether metformin affects mitochondrial respiratory capacity via a direct or indirect pathway, we studied mitochondrial respiration after incubating isolated mitochondria with 1 mM metformin for 5 min. We observed a 28% inhibition of Complex I-dependent respiratory capacity, whereas Complex II-dependent respiratory capacity was unaffected. Our findings thus imply that metformin inhibits mitochondrial respiration through Complex I via a direct pathway.

Apart from the effects of metformin on muscle mitochondrial function, we observed that *in vivo* muscle oxidative capacity was 25% lower in diabetic rats compared with lean control animals, independent of treatment regimen. However, relative mtDNA copy number and Complex I- and Complex II-dependent respiratory capacity were similar between diabetic and lean animals, which implies that neither a lower mitochondrial content nor an impairment of their *ex vivo* intrinsic function can account for the lower *in vivo* muscle oxidative capacity in diabetic rats. Instead, it suggests that in diabetic muscle the functioning of mitochondria in their natural cellular environment is impaired by factors that are not taken into account during the *ex vivo* measurements in isolated mitochondria, such as lipid-induced mitochondrial uncoupling [27].

The beneficial effects of metformin on glucose homeostasis are well established both in patient and animal studies [19], [34], [38]–[41]. However, in this study no changes in fasting plasma levels of glucose or insulin were observed in any of the animal groups after 2 weeks of treatment with metformin. It should be noted, though, that the therapy duration in our study was shorter than in the animal studies in which improved glucose tolerance was observed (typically 3 to 4 weeks) [34], which might explain why plasma parameters were unaffected in our study.

There are indications that the inhibition of Complex I contributes to metformin's therapeutic efficacy. It is well-known that metformin lowers blood glucose levels primarily by lowering glucose production in the liver, which is an ATP-dependent process. Therefore it is possible that the reduction of mitochondrial oxidative capacity underlies the mechanism through which metformin suppresses glucose release from the liver [39]. Moreover, there are several reports indicating that metformin promotes glucose uptake in peripheral tissues, thus contributing to its antihyperglycemic efficacy. This could be conciliated with its action on mitochondria by the “energy charge hypothesis” postulated by Brunmair *et al.* [4]. This hypothesis states that agents that interfere with Complex I-dependent cellular respiration affect enzymes like AMP-dependent protein kinase and hence induce a metabolic response, such as increased glucose uptake and glycolysis, to compensate for decreased ATP synthesis rates [3], [4].

In conclusion, we demonstrated that 2 weeks of treatment with metformin compromised *in vivo* and *ex vivo* muscle oxidative capacity in ZDF rats in a dose-dependent manner. Moreover, our finding that also *in vitro* incubation of isolated mitochondria with metformin lowers Complex I-dependent respiratory capacity supports the hypothesis that metformin inhibits Complex I via a direct pathway.
